# Supplementary material for: Distinct allosteric remodeling of HIV-1 Env dynamics on virions by gp41-directed antibodies reveals two modes of neutralization
Source: eLife. 2026 Oct 2;15:RP110887. doi: 10.7554/eLife.110887 (PMC13633075; doi:10.7554/eLife.110887)
Supplement: Supplementary file 2 [file elife-110887-supp1.docx]

S401–R542 structural perspective

| Conformational populations of Ab-incubated Env  on native virions | Curve fitting  *R^2^* | RMSE | **PT State** | **PC State** | **CO State** |
| --- | --- | --- | --- | --- | --- |
|  |  |  | **μ**: 0.55 ± 0.05 | **μ**: 0.28 ± 0.04 | **μ**: 0.10 ± 0.02 |
|  |  |  | **σ**: 0.15 ± 0.01 | **σ**: 0.09 ± 0.02 | **σ**: 0.07 ± 0.01 |
| Env_BG505_ | 0.9732 | 8.427e-4 | 59% ± 15% | 24% ± 14% | 17% ± 6% |
| Env_BG505_ + sCD4 + 17b | 0.9878 | 8.447e-4 | 28% ± 13% | 30% ± 13% | 42% ± 7% |
| Env_BG505_ + 8ANC195 | 0.9734 | 9.605e-4 | 45% ± 14% | 32% ± 15% | 23% ± 9% |
| Env_BG505_ + VRC34 | 0.9893 | 6.638e-4 | 34% ± 11% | 42% ±15% | 24% ± 9% |
| Env_BG505_ + PGT151 | 0.9895 | 6.858e-4 | 34% ± 14% | 47% ± 14% | 19% ± 5% |
| Env_BG505_ + DH511.2_K3 | 0.9890 | 7.149e-4 | 36% ± 14% | 45% ± 14% | 19% ± 6% |
| Env_BG505_ + VRC42 | 0.9865 | 8.237e-4 | 22% ± 10% | 53% ± 11% | 25% ± 5% |
| Env_BG505_ + 10E8.4 | 0.9877 | 8.375e-4 | 25% ± 16% | 51% ± 16% | 24% ± 8% |
| Env_BG505_ + iMab | 0.9756 | 8.503e-4 | 59% ± 16% | 27% ± 17% | 14% ± 11% |
| Env_BG505_ + 10E8.4/iMab | 0.9700 | 0.0012 | 28% ± 10% | 35% ± 12% | 37% ± 14% |
| Env_BG505_ + 10E8.4/iMab | 0.9700 | 0.0012 | 28% ± 10% | 35% ± 12% | 37% ± 14% |
| Env_BG505_ + 10E8.4 + sCD4 + 17b | 0.9910 | 8.471e-4 | 23% ± 16% | 33% ± 14% | 44% ± 6% |

N136–S401 structural perspective

| Conformational populations of Ab-incubated Env  on native virions | Curve fitting  *R^2^* | RMSE | **PT State** | **PC State** | **CO State** |
| --- | --- | --- | --- | --- | --- |
|  |  |  | **μ**: 0.10 ± 0.02 | **μ**: 0.60 ± 0.01 | **μ**: 0.32 ± 0.03 |
|  |  |  | **σ**: 0.08 ± 0.01 | **σ**: 0.15 ± 0.01 | **σ**: 0.11 ± 0.01 |
| Env_BG505_ | 0.9879 | 9.715e-4 | 42% ± 6% | 23% ± 11% | 35% ± 11% |
| Env_BG505_ + sCD4 + 17b | 0.9841 | 8.701e-4 | 29% ± 8% | 26% ± 13% | 45% ± 15% |

**Supplementary File S1**. Model-fitting statistics and parameters for FRET histograms.

FRET histograms acquired from two structural perspectives (top: S401 – R542; bottom: N136 – S401) of Ab-incubated Env on native virions were fitted independently using constrained three-state models. Each state was described by a Gaussian distribution *N* (*µ*, *σ*^2^) corresponding to PT, PC, and CO states. The mean (*µ*) and standard deviation (*σ*) parameters were determined separately for each perspective based on visual inspection of trajectories exhibiting state-to-state transitions and idealization of individual trajectories using multi-state Hidden Markov modeling. State probabilities are reported as mean ± s.e.m. Goodness-of-fit was assessed using *R*² and RMSE (root-mean-square error), with *R*² values approaching 1 and RMSE values approaching 0 indicating a high-quality fit.
