# Supplementary material for: Distinct allosteric remodeling of HIV-1 Env dynamics on virions by gp41-directed antibodies reveals two modes of neutralization
Source: eLife. 2026 Oct 2;15:RP110887. doi: 10.7554/eLife.110887 (PMC13633075; doi:10.7554/eLife.110887)
Supplement: Supplementary file 3 [file elife-110887-supp2.docx]

|  | **Heavy chain** | **Light chain** |
| --- | --- | --- |
| **PG16**  (VH+VL) | QEQLVESGGGVVQPGGSLRLSCLASGFTFHKYGMHWVRQAPGKGLEWVALISDDGMRKYHSDSMWGRVTISRDNSKNTLYLQFSSLKVEDTAMFFCAREAGGPIWHDDVKYYDFNDGYYNYHYMDVWGKGTTVTVSS | QSALTQPASVSGSPGQTITISCNGTSSDVGGFDSVSWYQQSPGKAPKVMVFDVSHRPSGISNRFSGSKSGNTASLTISGLHIEDEGDYFCSSLTDRSHRIFGGGTKVTVL |
| **PGT151**  (VH+VL) | RVQLVESGGGVVQPGKSVRLSCVVSDFPFSKYPMYWVRQAPGKGLEWVAAISGDAWHVVYSNSVQGRFLVSRDNVKNTLYLEMNSLKIEDTAVYRCARMFQESGPPRLDRWSGRNYYYYSGMDVWGQGTTVTVSS | DIVMTQTPLSLSVTPGQPASISCKSSESLRQSNGKTSLYWYRQKPGQSPQLLVFEVSNRFSGVSDRFVGSGSGTDFTLRISRVEAEDVGFYYCMQSKDFPLTFGGGTKVDLK |
| **8ANC195**  (VH+VL) | QIHLVQSGTEVKKPGSSVTVSCKAYGVNTFGLYAVNWVRQAPGQSLEYIGQIWRWKSSASHHFRGRVLISAVDLTGSSPPISSLEIKNLTSDDTAVYFCTTTSTYDKWSGLHHDGVMAFSSWGQGTLISVSAASTKGPSVF | DIQMTQSPSTLSASIGDTVRISCRASQSITGNWVAWYQQRPGKAPRLLIYRGAALLGGVPSRFSGSAAGTDFTLTIGNLQAEDFGTFYCQQYDTYPGTFGQGTKVEVKRTVAAPSVFIFPPSDEQLKSGT |
| **VRC34.01**  (VH+VL) | QEVLVQSGAEVKKPGASVKVSCRAFGYTFTGNALHWVRQAPGQGLEWLGWINPHSGDTTTSQKFQGRVYMTRDKSINTAFLDVTRLTSDDTGIYYCARDKYYGNEAVGMDVWGQGTSVTVSSX | DIQLTQSPSFLSASVGDKVTITCRASQGVRNELAWYQQKPGKAPNLLIYYASTLQSGVPSRFSATGSGTHFTLTVSSLQPEDFATYFCQHMSSYPLTFGGGTKVEIKX |
| **VRC42.01**  (VH+VL) | QVQLVQSGPEVKKPGSSLKVSCKASGGSFSTYTLSWVRQTPGQGLEWMGGIIPLLGLPNYAPKFQGRVTFSADTSTNTAYMEMSRLRFEDTAVYFCAREGAGWFGKPVGAMGYWGQGTTVTVSS | EIVLTQSPDTLSLSPGERASLSCRASQNVRNSNLAWYQHKPGQPPRLLIYGASSRASGIPGRFSGSGSGTDFTLTISRLEPEDFAVYYCQQYGGSFGTFGQGTKVEFR |
| **DH511.2**  (VH+VL) | QVQLVQSGGGLVKPGGSLTLSCSASGFFFDNSWMGWVRQAPGKGLEWVGRIRRLKDGATGEYGAAVKDRFTISRDDSRNMLYLHMRTLKTEDSGTYYCTMDEGTPVTRFLEWGYFYYYMAVWGRGTTVIVSS | DIVMTQSPSSVSASVGDRVTITCRASQNIRDYLNWYQHKPGGSPRLLIYAASTLQTGVPSRFSGSGSGNLFTLTITNLQPEDFATYYCQENYNTIPSLSFGQGTKVDIRX |
| **10E8.4** | EVRLVESGGGLVKPGGSLRLSCSASGFNFDDAWMTWVRQPPGKGLEWVGRISGPGEGWSVDYAESVKGRFTISRKNSKNTLYLEMNNVRTEDTGYYFCARTGKHYDFWSGYPPGEEYFQDWGQGTKVIVSSASTKGPSVFPLAPSSKSTSGGTAALGCLVKDYFPEPVTVSWNSGALTSGVHTFPAVLQSSGLYSLSSVVTVPSSSLGTQTYICNVNHKPSNTKVDKKVEPKSCDKTHTCPPCPAPEFEGGPSVFLFPPKPKDTLMISRTPEVTCVVVDVSHEDPEVKFNWYVDGVEVHNAKTKPREEQYNSTYRVVSVLTVLHQDWLNGKEYKCKVSNKALPASIEKTISKAKGQPREPQVYTLPPCRDELTKNQVSLWCLVKGFYPSDIAVEWESNGQPENNYKTTPPVLDSDGSFFLYSKLTVDKSRWQQGNVFSCSVLHEALHSHYTQKSLSLSPGK | ASELTQDPAVSVALKQTVTITCRGDSLRSHYASWYQKKTGQAPKLLFYGKNNRPSGVPDRFSGSASGNRASLTISGAQAEDDAEYYCSSRDKSGSRLSVFGGGTKLTVLSQPKAAPSVTLFPPSSEELQANKATLVCLISDFYPGAVTVAWKADSSPVKAGVETTTPSKQSNNKYAASSYLSLTPEQWKSHRSYSCQVTHEGSTVEKTVAPTECS |
| **iMab** | MEWSGVFMFLLSVTAGVHSQVQLQQSGPEVVKPGASVKMSCKASGYTFTSYVIHWVRQKPGQGLDWIGYINPYNDGTDYDEKFKGKATLTSDTSTSTAYMELSSLRSEDTAVYYCAREKDNYATGAWFAYWGQGTLVTVSSASTKGPSVFPLAPSSKSTSGGTAALGCLVKDYFPEPVTVSWNSGALTSGVHTFPAVLQSSGLYSLSSVVTVPSSSLGTQTYICNVNHKPSNTKVDKKVEPKSCDKTHTCPPCPAPEFEGGPSVFLFPPKPKDTLMISRTPEVTCVVVDVSHEDPEVKFNWYVDGVEVHNAKTKPREEQYNSTYRVVSVLTVLHQDWLNGKEYKCKVSNKALPASIEKTISKAKGQPREPQVYTLPPSRDELTKNQVSLTCLVKGFYPSDIAVEWESNGQPENNYKTTPPVLDSDGSFFLYSKLTVDKSRWQQGNVFSCSVLHEALHSHYTQKSLSLSPGK | MEWSGVFIFLLSVTAGVHSDIVMTQSPDSLAVSLGERVTMNCKSSQSLLYSTNQKNYLAWYQQKPGQSPKLLIYWANSTESGVPDRFSGSGSGTDFTLTISSVQAEDVAVYYCQQYYSYRTFGGGTKLEIKRTVAAPSVFIFPPSDEQLKSGTASVVCLLNNFYPREAKVQWKVDNALQSGNSQESVTEQDSKDSTYSLSSTLTLSKADYEKHKVYACEVTHQGLSSPVTKSFNRGEC |

**Supplementary File 2**. Amino acid sequences of the heavy and light chains of antibodies used in this study.
