# Supplementary material for: Distinct allosteric remodeling of HIV-1 Env dynamics on virions by gp41-directed antibodies reveals two modes of neutralization
Source: eLife. 2026 Oct 2;15:RP110887. doi: 10.7554/eLife.110887 (PMC13633075; doi:10.7554/eLife.110887)

Figure 1, Original membranes corresponding to Figure 1B. Specific lanes presented in the final manuscript are indicated with red box.
S, with supplements. NS, no supplements. M, protein standard markers. B, blank lane.

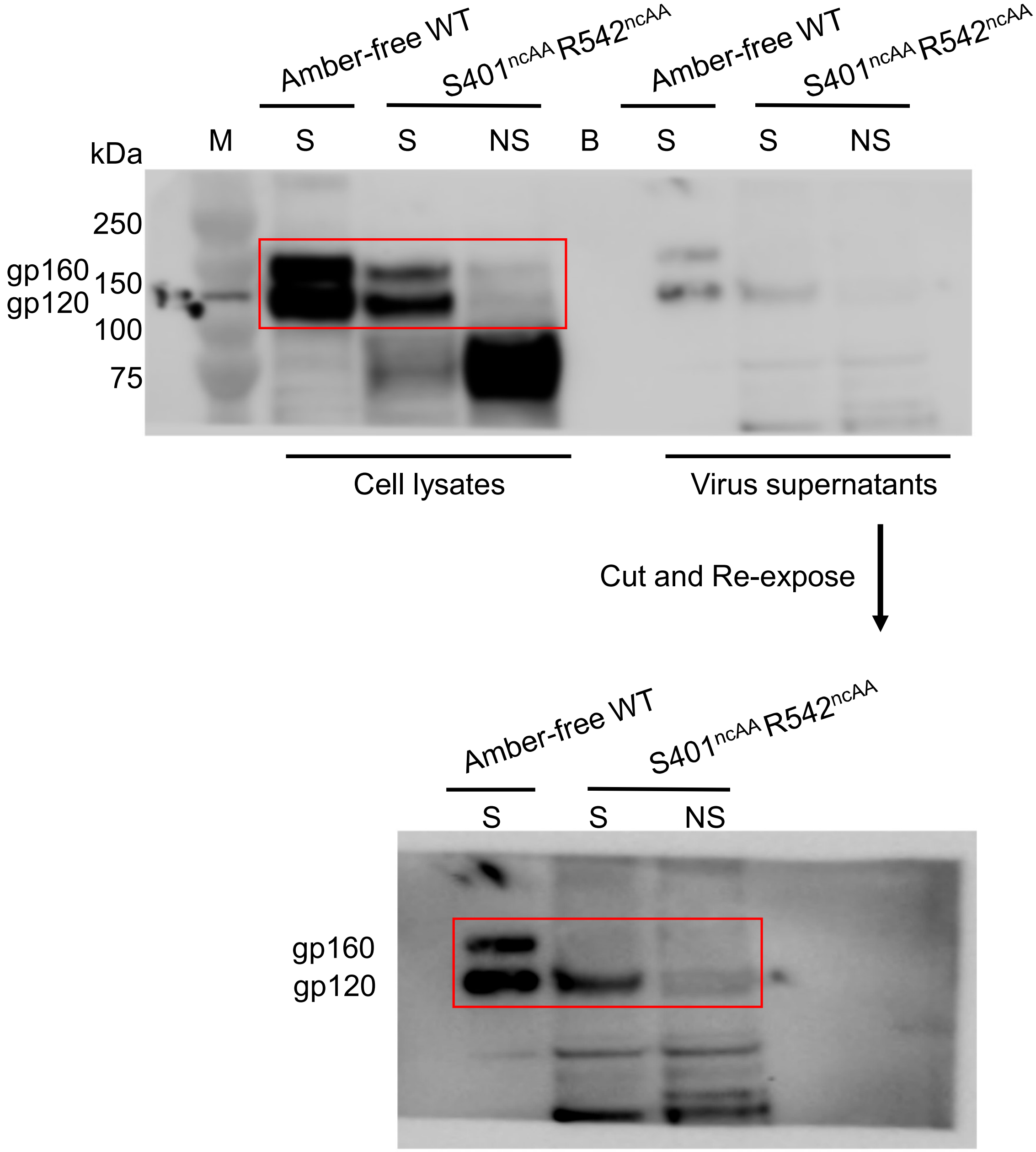

Supplement: Supplementary file 4 [file elife-110887-fig1-data1.zip › Figure 1 -source data 1.pdf]
