# Supplementary material for: Distinct allosteric remodeling of HIV-1 Env dynamics on virions by gp41-directed antibodies reveals two modes of neutralization
Source: eLife. 2026 Oct 2;15:RP110887. doi: 10.7554/eLife.110887 (PMC13633075; doi:10.7554/eLife.110887)
Supplement: Peer review history

# Editor report: eLife Assessment

**DOI:** 10.7554/eLife.110887.3.sa0

**Authors:** Volker Dötsch

**Affiliations:** Goethe University Frankfurt Germany

**Keywords:** Convincing, Important

This manuscript reports an **important** study in which the authors apply smFRET imaging to probe HIV-1 Env conformational dynamics in the presence of antibodies. Previous implementations of smFRET imaging of HIV-1 Env, which focus on gp120 conformation, have yielded limited information on antibodies that target gp41. Through the cutting-edge application of smFRET imaging, the study provides **convincing** insights into the mechanisms of action of relevant antibodies.

# Referee report: Reviewer #1 (Public review)

**DOI:** 10.7554/eLife.110887.3.sa1

The authors have considered a panel of antibodies that target epitopes at the gp120/gp41 interface (8ANC195 and PGT151), the fusion peptide in the gp41 domain (VRC34), and the MPER region of gp41 (DH511.2_K3 and VRC42). They also investigate 10E8.4/iMab, which is an engineered bispecific antibody that targets the MPER and the CD4 receptor. On a technical note, they have applied a double amber codon-readthrough strategy to incorporate the non-natural TCO*A amino acid, which gets labeled through click chemistry. This approach should result in less disruption of the native Env structure as compared to the peptide insertion previously used for smFRET imaging of Env. Furthermore, previous implementations of smFRET imaging of HIV-1 Env, which focus on gp120 conformation, have yielded limited information on antibodies that target gp41. Altogether, through the cutting-edge application of smFRET imaging, the study provides novel insights into the mechanisms of action of interesting and clinically relevant antibodies.

Comments on revised version:

The authors have nicely responded to all of my concerns. I have no further issues.

# Referee report: Reviewer #2 (Public review)

**DOI:** 10.7554/eLife.110887.3.sa2

Summary:

In this paper, Xu and co-workers unveil two distinct modes of neutralisation by gp41-targeted broadly neutralizing antibodies on HIV-1 Env. So far, it was unclear as to how the mechanism of neutralisation occurred for this subset of neutralising antibodies (that can target the fusion peptide or the membrane proximal external region of the gp41 subunit). Thanks to single-molecule FRET, the authors show that the majority of broadly neutralizing antibodies stabilize the closed Env conformation (named State 1 since the original work by Munro and colleagues PMID: 25298114). Interestingly, the bivalent 10E8.4/iMab stabilized in turn a CD4-bound open state of Env. The two modes of neutralization described for these antibodies show previously unknown allosteric mechanisms that stabilize closed and open Env conformation, stressing the importance of Env conformational dynamics and its efficiency during the process of fusion.

Strengths:

The article is well-written, and the figures fully depict the data in a convincing way. The authors have used smFRET, which is now established in the field as a good tool to assess Env dynamics.

Comments on revised version:

I am very happy with the comments, answers and the way the new manuscript is shaped after revision. I have no further questions or concerns.

# Author comment: Author response

**DOI:** 10.7554/eLife.110887.3.sa3

**Authors:** Wang Xu, Narendra Kumar Gonepudi, Junyu Liu, Yufan He, Revansiddha Katte, Ran Wang, Harry Baffour Awuah, Yang Han, Baoshan Zhang, Jian Yu, Bo Hu, David D Ho, Priyamvada Acharya, Peter D Kwong, Maolin Lu

**Affiliations:** The University of Texas Health Science Center at Tyler Tyler United States; The University of Texas Health Science Center at Tyler Tyler United States; The University of Texas Health Science Center at Tyler Tyler United States; The University of Texas Health Science Center at Tyler Tyler United States; The University of Texas Health Science Center at Tyler Tyler United States; The University of Texas Health Science Center at Houston Houston United States; The University of Texas Health Science Center at Tyler Tyler United States; The University of Texas Health Science Center at Tyler Tyler United States; Vaccine Research Center Bethesda United States; Columbia University New York United States; The University of Texas Health Science Center at Houston Houston United States; Columbia University New York United States; Duke University Durham United States; Columbia University New York United States; The University of Texas Health Science Center at Tyler Tyler United States

The following is the authors’ response to the original reviews.

**eLife Assessment:**

This manuscript reports an important study in which the authors apply smFRET imaging to probe HIV-1 Env conformational dynamics in the presence of antibodies. Previous implementations of smFRET imaging of HIV-1 Env, which focus on gp120 conformation, have yielded limited information on antibodies that target gp41. Through the cutting-edge application of smFRET imaging, the study provides convincing insights into the mechanisms of action of relevant antibodies.

We appreciate this positive assessment and thank the reviewers for their time and constructive comments. We have made the following changes in the revised manuscript to address all points raised by reviewers.

(1) Clarify the distinction between suppression efficiency and functional cost.

(2) Add controls: smFRET experiments in the presence of monovalent 10E8.4 and iMab individually.

(3) All of the smFRET population contour plots have been removed, as suggested.

(4) Repeat neutralization experiments of tagged viruses (carrying nc-AA-incorporated, amber-suppressed Env), add and compare infectivity profiles between before and after click-chemistry labeling of tagged viruses.

(5) Add a section (Complementary views from smFRET and structural studies) to the Discussion on how these approaches complement each other.

(6) Further clarify three prefusion conformational states identified by smFRET, the relation with previously identified States 1, 2, 3, and asymmetry, the heterogeneity of Env presentations and virion morphology, and the focus of this study.

Please find below our point-by-point responses to the public reviews and recommendations for the authors.

**Public Reviews:**

**Reviewer #1 (Public review):**

The authors have considered a panel of antibodies that target epitopes at the gp120/gp41 interface (8ANC195 and PGT151), the fusion peptide in the gp41 domain (VRC34), and the MPER region of gp41 (DH511.2_K3 and VRC42). They also investigate 10E8.4/iMab, which is an engineered bispecific antibody that targets the MPER and the CD4 receptor. On a technical note, they have applied a double amber codon-readthrough strategy to incorporate the non-natural TCO*A amino acid, which gets labeled through click chemistry. This approach should result in less disruption of the native Env structure as compared to the peptide insertion previously used for smFRET imaging of Env. Furthermore, previous implementations of smFRET imaging of HIV-1 Env, which focus on gp120 conformation, have yielded limited information on antibodies that target gp41. Altogether, through the cutting-edge application of smFRET imaging, the study provides novel insights into the mechanisms of action of interesting and clinically relevant antibodies.

Thank you for the positive comments!

In validating the functionality of the S401TAG/R542TAG Env, the authors performed infectivity assays and observed 20% infectivity as compared to wild-type (Figure S2A). However, the text equates this with "20% dual-amber suppression efficiency". This would benefit from some explanation. Why do the authors interpret infectivity as reporting on amber suppression efficiency, and not the functional cost of modifying Env, which is probably unavoidable? Or a combination of both? Is there data to suggest that 100% amber suppression would leave Env 100% functional? If so, this would be valuable to show. If not, the text should be clarified.

We acknowledge this concern and have clarified the distinction between suppression efficiency and functional cost in this revised manuscript. The observed reduction in infectivity does not translate into functional loss; instead, it more reflects the efficiency of suppression (one of the critical limitations of applying genetic code expansion in mammalian cells). To support the preservation of Env functionality, we performed dose-response neutralization experiments of tag-free and 100% dual-ncAA-incorporated Env virions by two trimer-specific neutralizing antibodies, which exhibited similar dose-dependent neutralization sensitivity (Fig. 1D), providing stronger validation than infectivity assays. We also compared infectivity between labeled and unlabeled virions and observed no significant difference (Fig. S3B).

We have previously discussed several limitations of amber suppression in mammalian cells when combined with smFRET viral systems (PMID: 38232732; PMID: 40716060) and, more recently, in our methodology chapters (PMID: 42349953; PMID: 42349954). In brief, orthogonal tRNA/aaRS pair–mediated amber suppression (reassigning/repurposing amber stop codons to non-canonical amino acids) of the introduced ambers in the target protein (Env in our case) must compete with the cellular translation system, particularly release factors that recognize amber codons and terminate translation. Readthrough of endogenous amber codons in virus-producing cells (in our case, HEK293T) can disrupt normal protein expression and virus production. Similarly, readthrough of pre-existing amber codons in HIV-1 ORFs other than the targeted ambers in Env can disrupt virus assembly, which we addressed by generating an amber-free provirus (PMID: 38232732). Introducing two amber codons into Env further reduces efficiency, as dual suppression requires two sequential successful suppression events within the same Env molecule.

The authors state that the contour plots in Figure 2E reveal "dynamic sampling" of the observed FRET states. Strictly speaking, as presented, the contour plots (and FRET histograms) provide no information on dynamics per se. They indicate only the relative thermodynamic stabilities of the FRET states; transitions between states are a matter of interpretation. The TDPs, shown later in Figure 5A, nicely display the dynamics. More importantly, interpretation of the contour plots is challenging, as some seem to suggest an evolution toward lower FRET states. This is especially evident in Figures 2F and 3D, which suggest that the system evolves into a stable 0.1-FRET state (CO) after about 3 sec. Unless the authors want to conclude something from this, I would suggest that they consider removing the contour plots, since their interpretations are fully supported by the FRET histograms alone.

We agree and have removed the contour plots, as they do not add meaningful information beyond what the histograms show.

The data indicating that Env conformation is manipulated by 10E8.4/iMab is interesting. If I understand correctly, 10E8.4/iMab is an engineered antibody with one Fab targeting MPER and the second Fab targeting CD4. In the absence of CD4, could the difference between 10E8.4/iMab and the other MPER antibodies be due to 10E8.4/iMab being monovalent with respect to MPER binding?

We appreciate this question. To address this, we have performed important controls: smFRET experiments in the presence of 10E8.4 and iMab individually in the absence of CD4. The results are shown in Fig. S9 in the revised manuscript, which indicates that 10E8.4 behaves similarly to other MPER-directed bNAbs we tested in this study, whereas iMab does not appear to affect the conformational populations of Env. The dual effect exerted by the bivalent 10E8.4/iMab is therefore very unexpected and thus interesting, as discussed in the Discussion section.

**Reviewer #2 (Public review):**

Summary:

In this paper, Xu and co-workers unveil two distinct modes of neutralisation by gp41targeted broadly neutralizing antibodies on HIV-1 Env. So far, it was unclear as to how the mechanism of neutralisation occurred for this subset of neutralising antibodies (that can target the fusion peptide or the membrane proximal external region of the gp41 subunit). Thanks to single-molecule FRET, the authors show that the majority of broadly neutralizing antibodies stabilize the closed Env conformation (named State 1 since the original work by Munro and colleagues PMID: 25298114). Interestingly, the bivalent 10E8.4/iMab stabilized in turn a CD4-bound open state of Env. The two modes of neutralization described for these antibodies show previously unknown allosteric mechanisms that stabilize closed and open Env conformation, stressing the importance of Env conformational dynamics and its efficiency during the process of fusion.

Strengths:

The article is well-written, and the figures fully depict the data in a convincing way. The authors have used smFRET, which is now established in the field as a good tool to assess Env dynamics.

We appreciate these positive comments!

Weaknesses:

(1) The limited controls on how click chemistry affects Env (as labelled Env HIV virions were not evaluated).

We agree. Our previous validation focused on ncAA-incorporated Env HIV-1 virions, but not the fluorescently labeled virions. To address this, we have added infectivity results for labeled virions after click-chemistry labeling, compared with those before labeling. We did not observe any measurable difference in infectivity (Fig. S3B), indicating that the labeling procedure does not impair viral infectivity.

We also attempted to perform dose-dependent neutralization after labeling. However, as anticipated in our provisional response, this remains technically challenging because the additional labeling and centrifugation steps substantially increase sample handling time, while the dual amber suppression system already limits virion production in cells. As a result, we were not able to obtain sufficiently robust datasets for this additional functional validation.

Nevertheless, we have previously demonstrated real-time tracking of single click-labeled Env virions during internalization and intracellular trafficking in live cells (PMID: 38232732), providing independent evidence that click-chemistry-labeled Env retains functional competence.

(2) Photobleaching of donor and acceptor molecules occurs right after 10sec exposure.

We acknowledge this limitation and have included it in the revision.

(3) Other limitations are well described in the corresponding section.

We appreciate this comment.

**Recommendations for the authors:**

**Reviewer #1 (Recommendations for the authors):**

As a means of clarifying the mechanism of 10E8.4/iMab, the authors might consider performing separate smFRET experiments in the presence of the normal 10E8.4 antibody and the normal iMab antibody (a negative control). Alternatively, they could consider imaging in the presence of the DH511.2_K3 and VRC42 Fabs (as opposed to full-length Ig) to make a cleaner comparison, although this may be less informative given the high concentrations of antibodies used.

We thank the reviewer for this excellent suggestion. To enable a direct comparison, we performed the most informative control by examining virus-associated Env in the presence of 10E8.4 alone and iMab alone. The corresponding smFRET results are presented in Fig. S9. We found that 10E8.4 behaves similarly to other MPER-directed antibodies, whereas iMab alone does not appear to have a notable effect on the conformational propensity of Env. For transparency and to facilitate future antibody design, we have also included the Fab region sequences of the antibodies in Table S2.

**Reviewer #2 (Recommendations for the authors):**

The article is well-written, the findings are of high interest for the community. The article should be shared once the points stated below are clarified and revised by the authors.

We appreciate this comment and the points raised by the reviewer and have revised the manuscript accordingly.

(1) In Figure 1C, the tomographic slices showing HIV-1 WT as compared to HIV-1 decorated with EnvBG505 S401ncAA R542nCAA are quite different morphologically. The micrographs chosen show a big particle with two capsids close to a smaller one without a capsid and at least in this plane bold (no Env incorporation) for the WT; whilst for the HIV-1 decorated with EnvBG505 S401ncAA R542nCAA no capsid is apparent in both particles, one (the right one) is very small and the right one does present a number of Envs but no apparent capsid is visible here. Please comment - perhaps it would be important to average the morphological traits of both and look at average diameter, average Env incorporation, morphology of the capsid, percentage of immature particles, capsid abnormalities (as the one shown in the upper micrograph).

We thank the reviewer for this thoughtful comment.

The tomograms in Fig. 1C were included to demonstrate the overall size and shape of the viral particles rather than to provide a quantitative structural comparison. HIV-1 viral particles are inherently heterogeneous, and the original slides were selected as representative examples. Following the reviewer's suggestion, we replaced the representative wild-type (Fig. 1C, top panel) and tagged virus (Fig. 1C, bottom panel) tomographic slides with those that better reflect the overall quality of each sample. To further address this concern, we refer the reviewer to the nanoparticle tracking analysis (NTA) shown in Fig. S3, which shows no significant difference in particle diameter between the wild-type and tagged viruses. In the revised manuscript, we now replace "morphology" with "shape" or "size," as these terms better reflect what our results can say.

We agree that a quantitative analysis of capsid morphology, Env spike incorporation, and the proportion of immature particles would be informative. However, such analyses would require a substantially larger cryoET dataset, which is beyond the scope of the present study; nevertheless, it is certainly in our interest to pursue a cryoET-focused study of EnvCA interactions, with Env complexed with 10E8.4/iMab. Our primary objective is to study Env conformational dynamics by smFRET rather than viral morphogenesis or capsid maturation, whose relationship to Env dynamics remains largely unexplored. It is also worth noting that the optimal particle populations for smFRET and cryoET differ. smFRET measures the conformational dynamics of individual Env trimers and therefore selectively analyzes virions containing a single dually labeled Env trimer, whereas cryoET structural analyses typically benefit from particles with higher Env spike densities. Therefore, the particle populations favored for the two techniques are not identical.

(2) In Figure 1D, there is a difference in neutralisation with PGT151 - how different are these two curves - how does the labelling affect neutralisation for bNAbs targeting gp41? Would it be possible to assess also the infectivity, fusion and neutralisation profiles of particles where the flurophores are included? This would be without diluting the Env for single particle analysis, but just to understand how harsh the organic reaction is and how it affects Env function (as all experiments and conclusions in the manuscript are based on labelled Env).

Again, we sincerely appreciate these questions, which have helped us improve the manuscript. Neutralization assays for the tagged viruses were performed using the ncAA-incorporated, amber-suppressed viruses, whereas the engineered wild-type is amber-free. The differences between these two dose-response curves in the original Fig. 1D are small and within the experimental variation routinely observed under even identical conditions (same virus and same bNAb). We have repeated these experiments, and the new results are shown in the revised Fig. 1D. Although minor variations remain, the overall neutralization profiles and IC50 values are highly consistent.

To assess whether the fluorophore labeling reaction affects Env functionality, as noted above, we have included infectivity results for labeled virions after click-chemistry labeling, compared with those before labeling. We did not observe any measurable difference in infectivity (Fig. S3B), indicating that the labeling procedure does not impair viral infectivity. We also attempted to perform dose-dependent neutralization after labeling. However, as anticipated in our provisional response, this remains technically challenging because the additional labeling and centrifugation steps substantially increase sample handling time, while the dual amber suppression system already limits virion production in cells. As a result, we were not able to obtain sufficiently robust datasets for this additional functional validation. Nevertheless, we have previously demonstrated real-time tracking of click-labelled Env virions during internalization and intracellular trafficking in live cells (PMID: 38232732), providing independent evidence that click-chemistry-labelled Env retains functional competence.

We believe that the unchanged infectivity of labeled viruses relative to their unlabeled counterparts, together with our previously observed real-time trajectories of click-labeled virions in live cells, provides strong evidence that our labeling strategy does not measurably impair Env function.

(3) In Figure 2E and 2G, the authors employ a three Gaussian fit approach to recover the three populations (pre-triggered - pre-fusion closed - CD4 bound open). Can you please relate these with State 1, 2 and 3 from the original article (PMID: 25298114). Comment on the possibility that more than three populations could be fitted and what this could mean - pre-triggered and partially open (one gp120 asymmetrically open) could occur? Could this labelling approach account for this asymmetry?

Thanks for this suggestion. In this study, we compared our results obtained using the gp120-gp41 structural axis with those obtained using the referenced gp120 V1-V4 structural axis to confidently assign the FRET-identified states to the previously reported three primary populations. The referenced axis is comparable to those used in the original article (PMID: 25298114) and later confirmed using the amber-click strategy (PMID: 38232732). We observe the same structural changes from these two distinct structural angles, as probed under ligand-free conditions (Fig. 2E and 2G) and CD4-triggered open conditions (Fig. 2F and 2H).

The pre-triggered state corresponds to State 1; the pre-fusion closed state corresponds to the symmetric State 2 (which the SOSIP-based soluble Env primarily adopts; PMID: 30971821); and the CD4-bound open state corresponds to the fully open State 3. The assignment of the FRET states observed from the gp120 V1-V4 structural axis to States 1, 2, and 3 was originally reported in two studies (PMIDs: 27795397 and 29561264). In the asymmetric trimer configuration, the State 2 FRET signal originates from the free protomer, while the other one or two protomers bind CD4 and adopt the open conformation (PMID: 29561264). The asymmetric intermediate (PMID: 29561264) was identified using a heterotrimer experimental design consisting of a mixture of wild-type and CD4-binding-incompetent D368R protomers, which was not used in the present study. Therefore, our labeling approach cannot unambiguously resolve this asymmetry.

Regarding the possibility of more than three populations, evidence from current and previous studies (PMIDs: 25298114, 27795397, 29561264, 38232732, 30971821) strongly supports the presence of three primary states of virus-associated Env, with additional substates that can be resolved under specific triggering conditions (PMIDs: 30974085, 41326374, 39640534). The assignment of such substates requires well-controlled experimental designs (PMIDs: 30974085, 41326374, 39640534).

We have related PT, PC, and CO to States 1, 2, and 3, and added comments on multiple states and asymmetry in the revised manuscript.

(4) When comparing smFRET with CryoET or structure, one can see that in smFRET there are always many potential conformations for big sub-populations of Env. Indeed, there is a trend, and the addition of bNAbs (Figure 4) clearly has an impact on increasing and stabilizing a particular state as defined by the authors (e.g. PT at 45% upon addition of 8ANC195, but also 32% PC and 23% CO). I assume that when analysing single particle CryoET or single virus CryoET, one needs to discard after template matching different scenarios that do not necessarily contribute to the highest resolution and this information is not always discussed. It would be interesting to address this in the discussion as the effect on Env dynamics of adding ligands (including CD4 and 17b) is not inducing in all Envs a drastic conformational change - this could be derived from the Ka of the ligands, but also from the intrinsic Env heterogeneity in both dynamics and architecture - I think that addressing these matters in the discussion could be of interest for the community. In this regard, the transition density plots are very helpful.

We completely agree and appreciate this insightful suggestion. We have expanded the Discussion to better address the complementary insights provided by smFRET and structural approaches. In single-particle cryoEM, we do not observe the full spectrum of Env conformations for technical reasons, not because particles are intentionally discarded to obtain only the highest-resolution structures. One reason is that open Env conformations are much more sensitive to radiation damage than closed Env. Likewise, ligand-free closed Env is more sensitive to radiation damage than a bNAb-stabilized closed Env. Thus, the outcome of an SPA cryo-EM study depends strongly on the biological question being addressed and the conformational state that is preferentially preserved under the experimental conditions. Although one could hypothetically collect much larger datasets to recover lower-abundance conformations, this would be both cost-prohibitive and unlikely to faithfully represent the relative conformational populations due to differential, conformation-dependent radiation damage.

We agree that the smFRET data highlight an important aspect of Env dynamics. Ligands, including bNAbs, CD4, and 17b, generally shift the conformational equilibrium toward particular states rather than driving all Env trimers into a single conformation. This likely reflects both differences in ligand binding properties and the intrinsic conformational heterogeneity of Env. We therefore believe that structural studies and smFRET provide complementary information. Structural methods resolve the molecular architecture of individual conformational states at atomic (by cryoEM) and near-atomic (by cryo-ET) levels, whereas smFRET quantifies their relative populations and dynamic interconversion. As the reviewer pointed out, the transition density plots are particularly valuable in illustrating these dynamic changes.

We have incorporated these points into the revised Discussion (Subtitle: complementary views from smFRET and structural studies in general).

(5) One of the very interesting findings of the paper is that the effect of bNabs (at least the ones tested) have an impact on the Env dynamics and how this shift can alter entry - therefore the structural view is perhaps less important - In spite of this, we still employ a structural jargon to refer to "Env open conformation stabilisation" for instance - even if the data shows that upon ligand exposure dynamics are still important but shifted. Please comment.

This is a great point. Our smFRET data show that bNAb binding generally shifts the conformational distribution toward and stabilizes particular Env states by lowering their free energy and increasing their occupancy. We have clarified that ligand-induced stabilization reflects a redistribution of the conformational ensemble while preserving the intrinsic dynamic nature of Env.
